# Assessment of α4, αv, β1 and β3 integrins expression throughout the implantation window phase in endometrium of a mouse model of polycystic ovarian syndromes

**Published:** 2014-10

**Authors:** Fatemeh Peyghambari, Mehri Fayazi, Saeid Amanpour, Mahnaz Haddadi, Samad Muhammadnejad, Ahad Muhammadnejad, Samira Abdolahpour, Mozhgan Enkesari, Zohreh Mazaheri

**Affiliations:** 1 *Department of Anatomical Sciences, Faculty of Medical Sciences, Islamic Azad University, Yazd Branch, Yazd, Iran.*; 2 *Department of Anatomical sciences, Medical Sciences Faculty, Tarbiat Modares University, Tehran, Iran.*; 3 *Vali-e-Asr Reproductive Health Research Centre, Tehran University of Medical Sciences, Tehran, Iran.*; 4 *Tumor Model Research Center, Cancer Institute of Iran, Tehran University of Medical Sciences, Tehran, Iran.*

**Keywords:** *Integrin*, *Endometrium*, *Implantation*, *Polycysticovariansyndromes*

## Abstract

**Background: **Endometrial integrin expression changes might be a reason for implantation failure in polycystic ovarian syndromes (PCOS).

**Objective** **:** Assessment of integrin genes and proteins expression upon endometrium in the PCOS experimental mouse model was the main goal of this study.

**Materials and Methods: **30 NMRI female mice were equally divided into control, experimental (PCOS; received estradiol valerate (40 mg/kg)) and sham group (received; olive oil). After 8 weeks, each group was hyper stimulated by 7 IU PMSG and then, after 48hrs, 7 IU HCG was injected. Vaginal plaque was checked. After 5 days, Progesterone and estradiol levels and endometrial tissues were investigated to evaluate of α4, αv, β1 and β3 integrins gene and protein by qPCR method and immunohistochemistry, respectively.

**Results: **Tissue samples were assessed and showed that level of progesterone was significantly decreased in PCOS group. Results of molecular part in the amount of αv, β3, β1 and α4 gene expressions showed a great difference in β3 and αv genes expressions between experimental groups. αv, β3, α4 and β1 proteins in the endometrial stroma in the control group were expressed, but they were not detected in PCOS group.

**Conclusion:** According to the results, integrins had different expression patterns in different areas of the endometrium; such as epithelial and stromal. It seems that in PCOS, this pattern has changed and the results might have a great influence on implantation failure. Therefore, this study suggests that a great attention to this problem may be essential in patients who are involved.

## Introduction

Polycystic ovary syndrome (PCOS) was the first illustrated by Stein and Leventhal in 1935. The major features of women with PCOS are polycystic ovaries and chronic anovulation, insulin resistance, obesity, irregular menstruation, infertility, and hirsutism (1-3). There is some evidences that the endocrinologic and metabolic imbalances in PCOS may have complex effects on the endometrium, take parting to the infertility and endometrial disorders observed in women with this syndrome (2, 4). The uterine receptivity disorder looks to be one of the most important factors in spontaneous abrasion cases such as PCOS patients, and cure to develop implantation rates is probable to be taken to this process (5). 

Researchers indicate that in women with PCOS, the αvβ3-integrin, HOXA-10, HOXA-11 and insulin-like growth factor (IGFBP-I) binding protein is down regulated throughout the secretory phase (3, 6-9). This is belief that integrins, cell adhesion molecules (CAMs), are good markers of endometrial receptivity and essential to cell contact. They represent a family of glycoproteins, formed by the two different non-covalent bonds: α and β subunits Integrins take part in cell matrix and cell-to-cell adhesion in many physiological events, such as embryological development, wound healing, immune and non-immune defense mechanisms and oncogenic transformation. A range of integrins has been expressed in the lumen of the glandular endometrial epithelium (10). Up regulation of α1β1, α4β1 and αvβ3 integrines has been explained in the endometrium in the mid-luteal phase, and the expression of subunit β3 demonstrated no increase before the 19th day, which has been suggested as an endometrial blastocyst receptor (11). 

They undergo hormonal effects, where high estrogen levels reduce the expression of integrins and increase the progesterone. Integrins have been expressed by the trophoblast at the time of implantation, demonstrating a sandwich model in embryonic attachment (12, 13). Despite technological progresses in assisted reproduction techniques, allowing the selection of top-quality embryos, the implantation rate is still low and has not increased adequately in recent decades (3, 14). Uterine receptivity has a key character in establishing the success of pregnancy and when changes, it may limit the success of assisted reproduction techniques and supply to infertility in certain gynecological diseases, such as PCOS (15). 

Literature concerns on the expression and regulation of α4β1 and αVβ3-integrin show that this protein may symbolize a marker for the human implantation process. The existing literature data are not very obvious if there is an endometrial dysfunction in PCOS, despite integrin action. Some studies show that the endometrial process, including cell proliferation, differentiation and response to biological effects, could be involved by the expression of integrins such as α4β1 and αVβ3-integrin, explaining in part the poor reproductive outcomes in this group of women (15).

We think that there is a lower expression of integrins in implantation window period in mice with PCOS than in normal mice. To understand this propose, we investigated gene and protein expression of α4β1 and αVβ3-integrin as some endometrial receptivity markers in the implantation window period of the experimental PCOS model, which were different from normal ones.

## Materials and methods

**Animals**

Thirty mature female mice were randomly divided to estradiol valerate (EV)-induced PCOS, sham and control groups as an experimental study (16). The mice were housed in a room under standard laboratory conditions (12 hr light/dark at 22^o^C) with free access to water and food at the cancer research center of Imam Khomeini hospital. The current study was conducted under the protocol approved by the animal experimentation committee at Islamic Azad University of Yazd animals’ laboratory. The EV group received 40 mg/kg body weight estradiol valerate by intramuscular [IM] (17). Sham group also received 100 µl of olive oil. All the groups were evaluated 60 days after the injection.

**Design** **of** **the** **study**

**Body** **weight** **measurement**

During the study, the body weight was registered, weekly. 

**Gonadal** **hormones** **assessment**

Serum was isolated from heart blood sample by centrifugation (2000 Rounds per min/10 min) in 3 mice from each group, 8 weeks post PCOS induction. 17-β estradiol and progesterone concentrations were evaluated by RIA method (a very sensitive in vitro assay technique used to measure concentrations of hormone levels) in Vali-e-asr hospital lab in the Imam Khomeini hospital.

**P** **olycystic ovaries evaluation**

Ovaries of each group were picked up after the treatment period (n: 3 mice), cleaned from adherent fat and connective tissue, and fixed in 10% formaldehyde buffer. Tissue processing and sectioning was down by routine histological techniques, then sections 5-6μm were stained with hematoxylin- eosin stain as described by Guyer, and observed under a light microscope [Olympus BX51, Germany] (18).

**Sampling of endometrial tissue** **during** **the** **implantation** **window period**

Each group was hyper stimulated by 7 IU PMSG (Folligon, Intervent, Australia) and after 48hr, 7 IU HCG (Sereno, Switzerland) was injected. Vaginal plaques were checked, and the pregnant mice were dislocated 5 days after the test. Endometrial tissue was picked up and divided into two parts. One part was put in 10% formalin buffer, and another part was kept in -70^o^C in order to extract RNA in RNA later for assessment of gene expression.

**Molecular assessment by quantitative polymerase chain reaction (qPCR)**

Total RNA was extracted from embryos in each group using QIAzol (QIAgen Germany) according to the manufacturer’s recommendations. To eliminate genomic contamination, RNA was treated with DNase I using a kit (EN0521; Fermentas). Concentrations of RNA were determined using a UV spectrophotometer (Eppendorff, Germany). The cDNAs were synthesized from 500 ng DNase-treated RNA samples with a RevertAid™ First Strand cDNA Synthesis kit (K1622; Fermentas, Germany) using oligo (dT) primers. For PCR reactions, primers were designed by the NCBI website and gene runner software and synthesized by Pishgam (Table I). PCRs were performed using Master Mix and SYBR Green in an Applied Biosystems, StepOne™ thermal cycler (Applied Biosystems, USA). The PCR program started with an initial melting cycle for 5 min at 95^o^C to activate the polymerase, followed by 40 cycles of melting (15 sec at 95^o^C), annealing (30 sec at 58^o^C) and extension (15 sec at 72^o^C). The quality of the PCR reactions was confirmed by melting curve analyses. Efficiency was determined for each gene using a standard curve (logarithmic dilution series). For each sample, the reference gene (β-actin) and target gene were amplified in the same run. Reference gene was approximately equal. The relative quantification of target genes, was normalized to a reference gene and determined using the method (19). 

**Protein assessment** **by** **immunohistochemistry method**

Endometrial samples were fixed in the formalin buffer. The tissues were sectioned for immunostaining analysis. We used antibodies specific for αv (1:100; Chemicon, UK), α4 (1:100; Abcam, UK), β1 (1:250; Abcam, UK) and β3 (1:250; Abcam, UK). HRP-labeled secondary rabbit antibody (BIO-IDEA, Iran) was used as a secondary antibody and DAB (Diaminibenzidine tetra hydrochloride hydrate, BIO-IDEA; Iran). Counter staining was done with hematoxylin dye. All of the methods for immunohistochemistry were done according to the manufacturer’s instructions. Images were captured using a light microscope equipped Olympus U-TVo.5 XC-3 camera. 

**Statistical** **analysis**

Data have been presented as the mean±SD. The results were analyzed by SPSS software (version 16.0) using One-way repeated measures analysis of variance (ANOVA) test between the groups. p˂0.05 were considered statistically significant, and it was followed by the Tukey post hoc test multiple.

## Results

**Figure 1:**
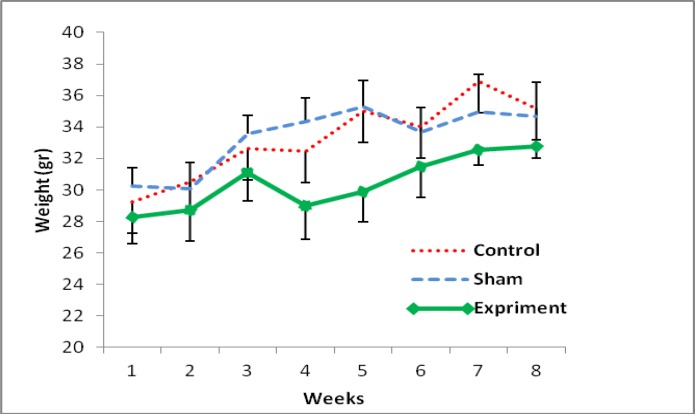
Mean of body weight changes after 8 weeks of PCOs induction in the experimental groups

Gaining weight in all groups was observed at the end of study, after 2 months of PCOS induction. Anyway among all groups, there was a significant difference between the first days of study and the last weeks. However, there was not any significant difference between groups (Figure 1). The amount of 17-β estradiol in the sera of control, sham and PCOS groups was 49.14±3.8, 41.17±4.1 and 133.11±5.6 pg/ml and that the progesterone level was 28±2.01, 35.12±1.72, 18.63±2.11 ng/ml, respectively. The level of 17-β estradiol increased significantly in EV-induced mice compared with the control and sham mice (p<0.01). Also, the level of progesterone decreased significantly in EV-induced mice compared with the other groups (p<0.04). 

**Figure 2:**
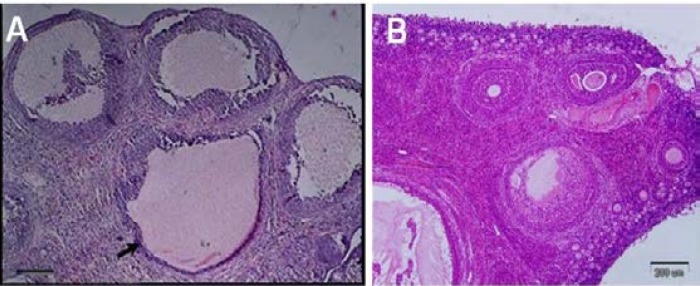
In the ovarian tissue, A; the cysts were mainly appeared by a single intramuscular dose of estradiol valerate, 40 mg/kg (H&E). B; ovarian tissue in the control group

**Figure 3:**
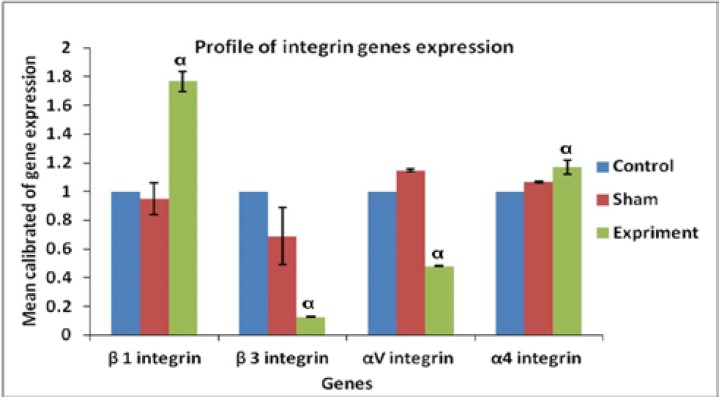
Real Time-PCR analysis. mRNA levels were normalized with respect to β-actin, chosen as an internal control and calibrated to control group. Profile of Integrin genes expression of endometer after using a single intramuscular dose of estradiol valerate, 40 mg/kg. Histograms show mean expression values (± SD; p< 0.05). α: significant difference with other groups in the same genes.

In the study of tissue sections, the presence of many cysts in ovaries full of cyct tissue in the PCOS induced groups was confirmed (Figure 2). Evaluations of molecular part in the amount of αv, β3, β1 and α4 gene expression showed that there was a great difference in gene expression between β3, αv and other groups. The results showed that β1 and α4 expressions in the endometrium of PCOS-induced mice were significantly (p≤0.001 and p≤0.046, respectively) increased whereas that of β3 and αv expression was significantly (p≤0.001) decreased compared with other groups; however, the expressions observed in the control and sham groups showed no significant difference in none of the integrin genes (Figure 3). 

**Figure 4:**
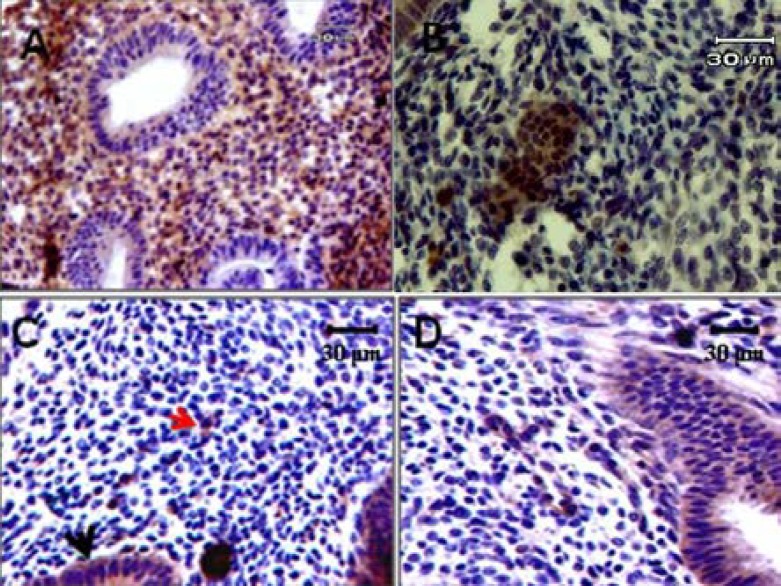
Immunostaining reactivity in endometrial tissue of normal mice. A; β3 Integrin expression, B; αV Integrin expression, C; α4 Integrin expression, D; β1 Integrin expression. Red arrow: stromal expression, Black arrow: Epithelial expression

**Figure 5:**
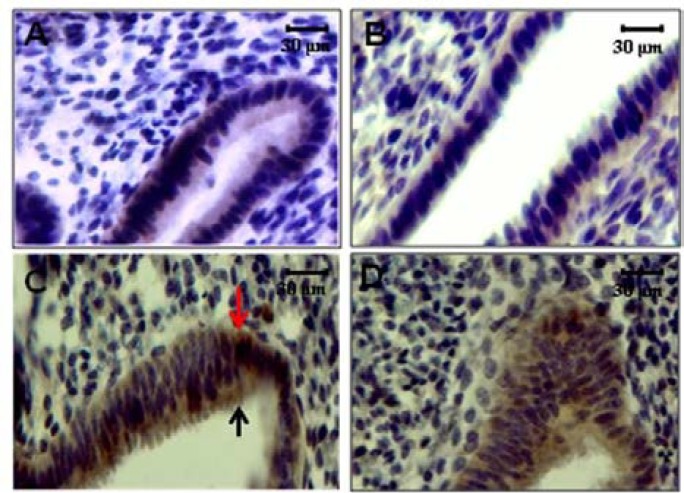
Immunostaining reactivity in endometrial tissue of PCOs model. A; β3 Integrin expression, B; αV Integrin expression, C; α4 Integrin expression, D; β1 Integrin expression. Red arrow: Basal epithelium expression, Black arrow: Luminal (apical) epithelium expression.

The αv, α4, β1 and β3 integrins were detected in the endometrium of all groups during the implantation window period. The expression of integrin molecules was as a diffuse pattern in apical and basal part of surface and glandular endometrial epithelium (Figure 4 & 5 A-D). The α4 subunit was most intense in the apical and basal membranes of the glandular uterine epithelial cells (Figure 4 & 5 C) and the αv subunit showed a diffuse pattern that was intense in the apical and basal membranes in both surface and glandular uterine epithelial cells (Figure 4 & 5 B). These two types of α subunit were not detected in the endometrial stroma of PCOS-induced mice during of the implantation window period. β1 integrin subunit exhibited a diffuse staining pattern, intensely positive in the membrane of glandular epithelial cells (Figure 4D). β3 integrin subunit was small intense in both surface and glandular epithelial cells of the endometrium of PCOS-induced mice, and it was not detected in the stroma (Figure 5A). All of integrin molecules and expression were restricted to the surface and glandular epithelium and was not detected in the stroma in PCOS-induced mice (Figure 5A-D).

**Table I:** Primer Used for real- time PCR

**Genes**	**Primer sequence**	**GenBank code**	**Tm (°C)**
β1 integrin	FOR: 5′- TGCCTACAACTCTCTTTCTTC-3′	NM_010578.2	58
	REV: 5′- TGGTTTCAGACTCCTTATTTG-3′		
β-actin	FOR: 5΄- TCCCTGGAGAAGAGCTACG-3΄	NM_001101	66.6
	REV: 5΄- GTAGTTTCGTGGATGCCACA-3΄		
β3 integrin	FOR: 5΄- TGGAAGAGCCTGAGTGTC-3΄	NM_016780	60
	REV: 5΄-CGGTAGGTGATATTGGTGAAG-3΄		
αV integrin	FOR: 5΄- GGAACAACGAAGCCTTAG-3΄	NM_008402	55
	REV: 5΄- GTATCCATCTCTGACTGC-3΄		
α4 integrin	FOR: 5׳- GAATCTCCTCCACCTACTCACAG -3׳	NM_010576	64
	REV: 5׳- CCAACGGCTACATCAACATATCC-3׳		

## Discussion

Understanding endometrial receptivity is an invasive subject in the fertility field in human. Thus, in this study, we used an animal PCOS model. An single dose injection of EV in the mouse produces an PCO condition. PCOS signs in mice, received 40 mg/kg estradiol valerate, were made after 8 weeks. The effect of estradiol valerate on hypophysis-hypothalamus pathway was observed after measuring of sexual hormone secretion. This plasma gonadotropin pattern indirectly was related to ovules, too and makes multiple small cysts. The cystic ovary produced by EV is similar in appearance to that seen in the human Stein-Leventhal condition, and thus provides a good model for the study of cystic ovarian disease (20-22).

Thus 8 weeks later, injection was used to assess hormone levels. Results have shown that a significant decrease in the progesterone level and a significant increase in the estrogen level in experimental groups. Luza *et al.* in 1995 and Brawer in 1978 made a hypothalamus-hypophysis pathway impairment by EV injection. This impairment could be a reason for change in the secretion of hypothalamic stimulators and consequently hypophysis secretion. This new pathway can affect the sexual hormone amount in blood serum (23-24).

We have demonstrated, for the first time, the expression of several integrin molecules in the endometrium of PCOS-induced mice, during the implantation window period using real-time RT-PCR in combination with immunohistochemistry techniques. Our observations, obtained by two techniques, demonstrated that there was not any sign of expression for integrins in the endometrial stroma of PCOS mice. These patterns of expression were correlated and synchronous well with the period when the serum progesterone concentration was increasing at the metestrus phase of the estrous cycle. The metestrus phase is like the secretory phase of the endometrium in human, and in this phase, the endometrium under the effect of circulatory progesterone undergoes some changes for reception of the embryo, and the implantation occurs in this phase (25).

It seems that the expression of these molecules in the endometrial stroma was regulated by progesterone while the estrogen had negligible effect in this regard. Regulation of integrin molecules expression is very complex and is hard to realize what regulates the expression of these during the implantation period. The expressions of epithelial αvβ3 integrin 6-8 days after ovulation, occurs with serum progesterone level increase, simultaneously (26). It has described that estrogen down-regulated αvβ3 expression, but no effect was observed for α4 or β1 integrin subunit (27). 

Down-regulation of α4 by progesterone and its up regulation by estradiol, also reported by some researcher in contrast to our study, in the blastocysts and the endometrium by a delayed-implantation mouse model (28). In this regards, some of studies showed that the expression of αv β3 integrin heterodimers not influenced by sex steroids in vivo (29-32). Lin *et* *al.* revealed that there is no correlation between the serum levels of steroid hormones and the αv and β3 integrins expression (33). These controversially outcomes might be due to some reasons like various types of integrin molecules, different protocols or experimental designs and types of species. 

Integrin cell adhesion molecules are one of the usual receptors upon endometerium surface in all mammals, and express in a cyclic pattern (27). We examined the profile of αv, β3, β1 and α4 expression in epithelial level and endometrial stroma in PCOS mice model. α4 subunit up regulated in the endometrium of the experimental mice groups than other molecules, and was expressed intense in the epithelium and stroma of the PCOS group. These findings can be a sign of roles and value of different types of integrins heterodimers like α4β3 α4β1, αvβ1, αvβ3 in different site of endometrium for making of contact between the embryo and endometrium during the implantation time. In this subject, Basak *et* *al* described the α4 integrin expression on the basement membrane of the endometrium might aid in adherent of the embryo to the uterine lumen (28).

 They have coated the α4 integrin by monoclonal antibodies in the endometrium of pregnant mice during implantation window and revealed the implantation failure in both normal as well as the delayed implantation mice (28). α4 epithelial subunit as a fibronectin receptor (α4β1) has a high expression in fertile and infertile women in the whole menstrual cycle. Epithelial α4 in fertile women increased in an implantation window period as similar as epithelial α4β1 expression in those who used anti-pregnancy pills (34-35). Stromal α4 is also seen in infertile women during the last proliferation period and the beginning of secretion (36). In the fertile women, the belief in molecular adherence expression pattern changed in the endometrial stroma is identified as unexplained infertility.

Epithelial β3 expression in fertile women is significantly increased in the implantation window period, while in infertile women this level is weakly expressed in the mid-secretion phase (37). Thus, a deficiency in the epithelial β3 expression can be a reason for infertility. In fertile women in the last part of the secretion, αv, α4, α1 and β3 are secreted. Molecular importance of this change in infertile women is not known (34). 

αv expression, which is a vitronectin receptor (αvβ3), has a different expression pattern related to its molecule, i.e. β3 (38). Integrin αv only expresses in last secretion phase in the epithelium of infertile women (36). This protein in women's epithelium and in Ishikawa cells is also shown. αv in addition to β3 can be expressed along other β subunits such as β1, β5 and β8 (34-35). 

A decrease in stromal β1 and α1 in unexplained infertile women is seen that can cause a deficiency in trophoblast linking with stromal cells. Regulation of β1 and α1 and their function during the implantation period in human is actually unidentified. The function of the endometrial steroid hormones by some growth factors and cytokines may result in integrin expression depended on the cycle (39). So, based on other studies, it was revealed that hormonal fluctuations due to cystic ovarian syndrome and can regulate Integrin cell adhesion molecules involved in endometrium and embryo attachment. Furthermore, expression out of the cycle in these molecules can directly affect fertility in patients who are being treated.

## Conclusion

The present study shows that in mouse with PCOS, αv and β3 integrin expression in the endometrial tissue was significantly lower than normal mouse. Additionally, in these animal model, a high expression of α4 and β1, involved integrins in the implantation process in a cyclic way during the whole period, could exist. Although this expression in different areas of the endometrium such as the epithelial and stromal, had a different expression pattern in investigated groups, but it seems that in PCOS, this pattern had changed and the results had a great effect on implantation failure. 
